# The clinicopathologic characteristics of familial and sporadic papillary thyroid carcinoma in Turkish patients

**DOI:** 10.3906/sag-1907-94

**Published:** 2020-04-09

**Authors:** Havva SEZER, Mehmet Onur DEMİRKOL, Dilek YAZICI, Yersu KAPRAN, Faruk ALAGÖL

**Affiliations:** 1 Department of Endocrinology and Metabolism, Faculty of Medicine, Koç University, İstanbul Turkey; 2 Department of Nuclear Medicine, Faculty of Medicine, Koç University, İstanbul Turkey; 3 Department of Pathology, Faculty of Medicine, Koç University, İstanbul Turkey

**Keywords:** Familial, sporadic, papillary thyroid cancer

## Abstract

**Background/aim:**

The aim of this study is to investigate clinicopathologic features of familial papillary thyroid carcinoma (fPTC) and compare them with sporadic papillary thyroid carcinoma (sPTC) in Turkish patients. A retrospective analysis of the papillary thyroid carcinoma (PTC) cases, with or without family history with a follow-up around 10 years was performed.

**Materials and methods:**

A series of patients with fPTC (82 fPTC families with 146 affected individuals) were compared with patients with sPTC (n = 112). The clinicopathologic features [(age, gender, histologic subtype, tumour size, bilaterality, multifocality, extrathyroidal extension (ETE), lymph node metastasis (LNM)] and treatment procedures (lymph node dissection, radioactive iodine ablation), and the outcomes like recurrences in the neck region, distant metastasis, and the need for reoperation were compared between the groups.

**Results:**

When the groups were compared, there was no significant difference in age (P = 0.449), and tumour size (P = 0.898) between familial and sporadic PTC patients. fPTC group had a significantly higher risk of male gender (P=0.001), bilaterality (P = 0.004), multifocality (P = 0.011), LNM (P = 0.013), ETE (P = 0.040), and distant metastasis (P ≤ 0.0001) than the sPTC group. However, recurrence rate was similar between the 2 groups (P = 0.436).

**Conclusion:**

The results of this study confirms a more aggressive nature in fPTC patients, in terms of bilaterality, multifocality, ETE, LNM, and distant metastasis, compared to sPTC patients in Turkish population.

## 1. Introduction

Well differentiated thyroid carcinoma is the most common endocrine related cancer (80%–90% papillary, 10%–15% follicular) [1]. The majority of papillary and follicular thyroid carcinomas are sporadic, but some are inherited (familial or associated with hereditary cancer syndromes) [1]. These inheritable cancers constitute approximately 8% of all nonmedullary thyroid cancers [1]. PTC is the most common histological subtype, followed by PTC follicular variant, follicular thyroid carcinoma, and very rarely seen anaplastic carcinoma [2]. Familial nonmedullary thyroid cancers can be divided into 2 clinicopathological groups. The first group includes several syndromes that are associated with an increased risk of thyroid cancer like Cowden syndrome, familial adenomatous polyposis, Werner syndrome, and Carney complex. These may have an increased risk of benign thyroid disease as well [3]. The second group includes familial syndromes characterized by predominance of PTC, such as pure fPTC, fPTC associated with papillary renal cell carcinoma, and fPTC with multinodular goiter [3]. fPTC can be diagnosed when 2 or more first degree family members are affected with thyroid cancer of follicular cell origin including the index patient with the exclusion of other familial cancer syndromes [4]. When there are 2 affected family members with thyroid cancer, the probability that the affected members have a familial syndrome is reported to be 38% [5]. However, in families with 3 or more affected family members with thyroid cancer, the probability increases to more than 96% [5]. fPTC was first reported in 1955 by Robinson and Orr in monozygotic twins [6]. fPTC is genetically heterogeneous and multigenic between different families [7]. Genes responsible for fPTC have not been identified yet [7]. Nowadays, it is believed that, fPTC is due to a combination of genetic and environmental factors [7]. 

Most authors agree that fPTC is more aggressive than sPTC. This aggressiveness is characterized by multicentricity, larger tumour size, ETE, vascular invasion, and lymph nodal involvement [4,8–17]. Since there is also contradictory data, it remains controversy [1,18–21].

Turkey is in a region that is with an increased risk of thyroid cancer. Iodine deficiency and being in close proximity to the Chernobyl accident have increased the incidence of thyroid cancer in Turkey [22]. There is no data determining the aggressiveness of fPTC in Turkish patients. Data from some countries as Italy, China, Japan, and Greece indicate increased aggressiveness on the side of fPTC [10,11,23,24]. However, a recent study from Bulgaria did not demonstrate any difference in the aggressiveness of fPTC and sPTC [20]. 

The aim of this study is to investigate the clinical and pathological features of fPTC and compare them with sPTC in Turkish patients.

## 2. Materials and methods

All the patients included in this study were diagnosed and were treated for PTC between 2001 and 2015. The study was a retrospective study, and all procedures in this study were approved by our hospital’s ethics committee (Ethical approval number 2015.104.IRB2.041 with a date of 23 June 2015). Follow-up data information was obtained from hospital records. Information about the family history of thyroid cancer was obtained from the patients’ records. Patients with a positive family history were classified as fPTC when at least 2 first degree family members were affected without any other familial cancer syndromes. There were no patients with other familial cancer syndromes in our study population. To determine whether fPTC had aggressive behaviour, the clinical and pathological features such as age at diagnosis, gender, tumour size, histological variant, multifocality, bilaterality, ETE, vascular invasion, LNM, distant metastasis, recurrent disease, and treatment characteristics (central lymph node dissection, remnant ablation) were compared between sPTC and fPTC patients. 

This study included a total of 258 patients; 146 patients with fPTC, and aged matched 112 sPTC among patients. All patients of the study had undergone total thyroidectomy. Lateral compartment lymph node dissection was performed in patients with biopsy-proven metastasis. Radioactive iodine (RAI) treatment had been performed according to tumour size of >10 mm and the presence of multifocality, ETE, LNM, and distant metastasis. Thyroid hormone treatment had been initiated after the remnant ablation to decrease serum TSH to subnormal level. In patients treated with total thyroidectomy, measurement of serum thyroglobulin (Tg), anti-Tg antibodies during therapy with levothyroxine and neck ultrasonography had been performed 6 months after surgery and then annually.

Persistent/recurrent disease was defined: 

1) When stimulated Tg exceeded 2 ng/ml in patients who underwent total thyroidectomy

2) Metastatic lymph nodes were detected by neck ultrasound and confirmed by fine-needle aspiration cytology

3) Positive 131-I-whole body screening (WBS)

The median postoperative follow-up duration was 10 years (min: 3 years, max: 17 years). 

### 2.1. Statistical analysis

Statistical analysis was performed with a SPSS software package version 25 (IBM Corp., Armonk, NY, USA). Categorical variables were expressed as number while continuous variables were expressed as mean ± standard deviation. Pearson chi-square (*χ*2) test was used to compare groups regarding categorical variables. Continuous variables were compared with student t-test. The results were evaluated at P < 0.05 significance level with a 95% confidence interval.

## 3. Results

The mean age at diagnosis was 43.3 ± 13.7 years in patients of fPTC, and 41.2 ± 10.5 years in patients of sPTC. The male to female ratio was greater in the fPTC group compared to sPTC group (in fPTC group: 46/100, in sPTC group: 20/92) (P = 0.001). The mean tumour diameter was 11.1 ± 7.5 mm in patients of fPTC, and 11.6 ± 7.9 mm in patients of sPTC. There were no significant differences in terms of age at diagnosis (P = 0.449) and tumour size (P = 0.898) between the 2 groups. 

fPTC group had a significantly higher risk of bilaterality (P = 0.004), multifocality (P = 0.011), ETE (P = 0.040), LNM (P = 0.013), and distant metastasis (P ≤ 0.0001) than the sPTC group (as shown in Table 1). All of the metastasis were to lung in fPTC patient group. The histological types in each group were comparable (P = 0.817). There were 3 patients with tall cell variant and 3 patients with diffuse sclerozing variant in fPTC group. On the other hand lymphatic and vascular invasion were more prominent in the sPTC group compared to the fPTC group (P ≤ 0.0001, P = 0.011, respectively). fPTC group was more prone to have coexistence with lymphocytic thyroiditis compared with the sPTC group (P ≤ 0.0001). 

**Table 1:** Clinicopathological charatcteristics and prognostic outcomes of the patients. Familial versus sporadic group.

	fPTC (n = 146)	sPTC (n = 112)	P-value
Age ≥ 45 years	62 (42.5%)	36 (32.1%)	0.449
Age < 45 years	84 (57.5%)	76 (67.9%)	
Tumour size > 10 mm	74 (50.7%)	59 (52.7%)	0.898
Tumour size ≤ 10 mm	72 (49.3%)	53 (47.3%)	
Male	46 (31.5%)	20 (17.9%)	0.001
Female	100 (68.5%)	92 (82.1%)	
Multifocality	71 (48.6%)	39 (34.8%)	0.011
Bilaterality	46 (31.5%)	29 (25.8%)	0.004
Classical variant	83 (56.8%)	66 (59.0%)	0.817
Nonclassical variant	63 (43.2%)	46 (41.0%)	
Lymph node metastasis	34 (23.2%)	23 (20.5%)	0.013
Lymphatic invasion	13 (8.9%)	33 (29.4%)	<0.0001
Vascular invasion	12 (8.2%)	12 (10.7%)	0.011
ETE	28 (19.1%)	21 (18.7%)	0.040
Distant metastasis	5 (3.4%)	0 (0%)	<0.0001
Lymphocytic thyroiditis	71 (48.6%)	41 (36.6%)	<0.0001
RAI ablation	110 (75.3%)	78 (69.6%)	0.007
Recurrence/re-operation	19 (13.0%)	11 (9.8%)	0.436	ETE: Extrathyroidal extension, RAI: Radioactive iodine.			

The number of patients that had RAI ablation therapy were higher in the fPTC group compared to the sPTC group (P = 0.007). The recurrences were at the lymph nodes in the central or lateral regions in both groups. All of the recurrences were treated with reoperations (as central or lateral region dissections). Thus, the reoperation rates indicate the recurrence rates. The recurrence rates were similar between the groups (P = 0.436). There was no mortality in follow up period around 10 years in both of the 2 groups. 

The clinicopathological characteristics were also compared according to the number of affected relatives in the familial group (as shown in Table 2). One hundred and forty six fPTC patients represented 82 families. We divided the fPTC patient group into 2 subgroups: 1) 122 patients of the 68 families with fPTC had 2 affected family members, and 24 patients of the 14 families with fPTC had 3 or more affected family members. The mean age at diagnosis was 43.6 ± 14.1 years in patients with 2 affected family members, and 41.4 ± 10.6 years in patients with ≥ 3 affected family members. The mean tumour diameter was 10.0 ± 7.7 mm in patients with 2 affected family members, and 11.4 ± 5.7 mm in patients with ≥ 3 affected family members. There were no significant differences in age at diagnosis (P = 0.502) and tumour size (P = 0.104) between the 2 groups. The number of microcarcinomas were more in the group with 2 affected family members compared to ≥ 3 affected family members. The tumour subtype was not different between the groups (P = 0.616). The incidence of multifocality and having LNM were similar between the groups (P = 0.208, P = 0.149, respectively). Vascular invasion and ETE were more prominent in the group of 2 affected family members compared to the group with ≥ 3 affected family members (P = 0.039, P = 0.0001, respectively). The incidence of bilaterality, and distant metastasis in the group with ≥ 3 affected family members were significantly higher than in the group with 2 affected family members (P = 0.036, P = 0.008, respectively). More patients in the group with ≥ 3 affected family members had lymphocytic thyroiditis compared to the other group (P = 0.026). RAI ablation treatment was performed more frequently in the group with 2 affected family members compared to the other group (P = 0.009). Finally the need for reoperation was similar between the groups (P = 0.992). 

**Table 2:** Clinicopathological characteristics and prognostic outcomes of fPTC patients according to the number of affected family members. Two versus 3 affected members.

	Family = 2 (n = 122)	Family ≥ 3 (n = 24)	P-value
Age ≥ 45 years	52 (42.7%)	10 (41.7%)	0.502
Age < 45 years	70 (57.3%)	14 (58.3%)	
Tumour size > 10 mm	56 (45.9%)	18 (75.0%)	0.104
Tumour size ≤ 10 mm	66 (54.1%)	6 (25.0%)	
Female	83 (68.0%)	17 (70.8%)	0.061
Male	39 (32.0%)	7 (29.2%)	
Multifocality	59 (48.3%)	12 (50.0%)	0.208
Bilaterality	37 (30.3%)	9 (37.5%)	0.036
Classical variant	70 (57.3%)	13 (54.1%)	0.616
Nonclassical variant	52 (42.7%)	11 (45.9%)	
Lymph node metastasis	26 (21.3%)	8 (33.3%)	0.149
Lymphatic invasion	10 (8.2%)	3 (12.5%)	0.013
Vascular invasion	11 (9.0%)	1 (4.2%)	0.039
ETE	25 (20.4%)	3 (12.5%)	0.0001
Distant metastasis	3 (2.5%)	2 (8.3%)	0.008
Lymphocytic thyroiditis	58 (47.5%)	13 (54.1%)	0.026
RAI ablation	95 (77.8%)	15 (62.5%)	0.009
Recurrence/re-operation	16 (13.1%)	3 (12.5%)	0.992
ETE: Extrathyroidal extension, RAI: Radioactive iodine.			

The sPTC group was compared with the fPTC group of 2 affected family members (as shown in Table 3). The incidence of multifocality (P = 0.003), bilaterality (P = 0.036), ETE (P = 0.026), distant metastasis (P ≤ 0.0001), and lymphocytic thyroiditis (P ≤ 0.0001) in families with 2 affected family members were significantly higher than those of the sPTC group. 

**Table 3:** Clinicopathological characteristics and prognostic factors of sPTC versus fPTC with 2 affected members.

	Family = 2 (n = 122)	sPTC (n = 112)	P-value
Age ≥ 45 years	52 (42.7%)	36 (32.1%)	0.449
Age < 45 years	70 (57.3%)	76 (67.9%)	
Tumour size > 10 mm	56 (45.9%)	59 (52.7%)	0.681
Tumour size ≤ 10 mm	66 (54.1%)	53 (47.3%)	
Female	83 (68.0%)	92(82.1%)	0.019
Male	39 (32.0%)	20 (17.9%)	
Multifocality	59 (48.3%)	39 (34.8%)	0.003
Bilaterality	37 (30.3%)	29 (25.8%)	0.036
Classical variant	70 (57.3%)	66 (59.0%)	0.788
Nonclassical variant	52 (42.7%)	46 (41.0%)	
Lymph node metastasis	26 (21.3%)	23 (20.5%)	0.546
Lymphatic invasion	10 (8.2%)	33 (29.4%)	<0.0001
Vascular invasion	11 (9.0%)	12 (10.7%)	0.566
ETE	25 (20.4%)	21 (18.7%)	0.026
Distant metastasis	3 (2.5%)	0 (0%)	<0.0001
Lymphocytic thyroiditis	58 (47.5%)	41 (36.6%)	<0.0001
RAI ablation	95 (77.8%)	78 (69.6%)	0.005
Recurrence/re-operation	16 (13.1%)	11 (9.8%)	0.829
ETE: Extrathyroidal extension, RAI: Radioactive iodine.			

When clinopathological characteristics of the sPTC group was compared with the fPTC group of 3 or more affected family members (as shown in Table 4), ETE was more evident in the sporadic group (P = 0.039) and distant metastasis was more in the fPTC group (P ≤ 0.0001). RAI was given more in the sPTC group compared to the fPTC group (P = 0.005).

**Table 4:** Clinicopathological characteristics and prognostic factors of sPTC versus fPTC with 3 or more affected members.

	Family ≥ 3 (n = 24)	SPTC (112)	P-value
Age ≥ 45 years	10 (41.7%)	36 (32.1%)	0.800
Age < 45 years	14 (58.3%)	76 (67.9%)	
Tumour size > 10 mm	18 (75.0%)	59 (52.7%)	0.991
Tumour size ≤ 10 mm	6 (25.0%)	53 (47.3%)	
Female	17 (70.8%)	92(82.1%)	0.076
Male	7 (29.2%)	20 (17.9%)	
Multifocality	12 (50.0%)	39 (34.8%)	0.290
Bilaterality	9 (37.5%)	29 (25.8%)	0.384
Classical variant	13 (54.1%)	66 (59.0%)	0.252
Nonclassical variant	11 (45.9%)	46 (41.0%)	
Lymph node metastasis	8 (33.3%)	23 (20.5%)	0.254
Lymphatic invasion	3 (12.5%)	33 (29.4%)	0.079
Vascular invasion	1 (4.2%)	12 (10.7%)	0.407
ETE	3 (12.5%)	21 (18.7%)	0.039
Distant metastasis	2 (8.3%)	0 (0%)	<0.0001
Lymphocytic thyroiditis	13 (54.1%)	41 (36.6%)	0.022
RAI ablation	15 (62.5%)	78 (69.6%)	0.005
Recurrence/re-operation	3 (12.5%)	11 (9.8%)	0.966
ETE: Extrathyroidal extension, RAI: Radioactive iodine.			

When the patients with the classical subtype of PTC in both groups were compared in terms of the clinopathological characteristics (as shown in Table 5), multifocality (P = 0.046), ETE (P < 0.0001), distant metastasis (P < 0.0001) and the presence of lymphocytic thyroiditis (<0.0001) were more evident in the fPTC. Lymphatic invasion was more prominent in the sPTC group (P = 0.041).

**Table 5:** Clinicopathological characteristics and prognostic outcomes of the patients with classical variant. Familial versus sporadic group.

	fPTC (n = 83)	sPTC (n = 66)	P-value
Age ≥ 45 years	31 (37.4%)	20 (30.3%)	0.742
Age < 45 years	52 (62.6%)	46 (69.7%)	
Tumour size > 10 mm	36 (43.4%)	34 (51.5%)	0.743
Tumour size ≤ 10 mm	47 (56.6%)	32 (48.5%)	
Female	55 (66.3%)	53 (80.3%)	0.492
Male	28 (33.7%)	13 (19.7%)	
Multifocality	46 (55.4%)	14 (21.2%)	0.046
Bilaterality	29 (34.9%)	13 (19.6%)	0.279
Lymph node metastasis	22 (26.5%)	20 (30.3%)	0.458
Lymphatic invasion	10 (12.0%)	25 (37.8%)	0.041
Vascular invasion	9 (10.8%)	7 (10.6%)	0.935
ETE	19 (22.8%)	12 (18.1%)	<0.0001
Distant metastasis	2 (2.4%)	0 (0%)	<0.0001
Lymphocytic thyroiditis	46 (55.4%)	31 (46.9%)	<0.0001
RAI ablation	69 (83.1%)	51 (77.2%)	0.851
Recurrence/re-operation	10 (12.0%)	2 (3.0%)
0.467	ETE: Extrathyroidal extension, RAI: Radioactive iodine.			

When the patients with the nonclassical subtype of PTC in both groups were compared in terms of the clinopathological characteristics (as shown in Table 6), distant metastasis (P < 0.0001), and the presence of lymphocytic thyroiditis (<0.0001) were more evident in the fPTC group.

**Table 6:** Clinicopathological characteristics and prognostic outcomes of the patients with nonclassical variant. Familial versus sporadic group.

	fPTC (n = 63)	sPTC (n = 46)	P-value
Age ≥ 45 years	31 (49.2%)	16 (34.8%)	0.163
Age < 45 years	32 (50.8%)	30 (65.2%)	
Tumour size > 10 mm	38 (60.3%)	25 (54.3%)	0.566
Tumour size ≤ 10 mm	25 (39.7%)	21 (45.7%)	
Female	45 (71.4%)	39 (84.7%)	0.964
Male	18 (28.6%)	7 (15.3%)	
Multifocality	25 (39.6%)	25 (54.3%)	0.468
Bilaterality	17 (26.9%)	16 (34.7%)	0.416
Lymph node metastasis	12 (19.0%)	3 (6.5%)	0.719
Lymphatic invasion	3 (4.7%)	8 (17.3%)	0.749
Vascular invasion	3 (4.7%)	5 (10.8%)	0.162
ETE	9 (14.2%)	9 (19.6%)	0.057
Distant metastasis	3 (4.7%)	0 (0%)	<0.0001
Lymphocytic thyroiditis	25 (39.6%)	10 (21.7%)	<0.0001
RAI ablation	41 (65.0%)	27 (58.7%)	0.235
Recurrence/re-operation	9 (14.2%)	9 (19.5%)
0.057	ETE: Extrathyroidal extension, RAI: Radioactive iodine.			

## 4. Discussion

The clinical behaviour of fPTC is still controversial. Some studies found that the biological behaviour of fPTC tends to be more aggressive than sPTC [8,23,25], but others have claimed that there are no differences between the 2 groups [18–21,26]. In our study, fPTC and sPTC were similar based on recurrence rate, but some differences were observed in the clinicopathological features. Multifocality, bilaterality, ETE, LNM, and distant metastasis were significantly higher in the fPTC patients compared to the sPTC patients. To our knowledge, this is the first study to investigate the clinocopathological characteristics of fPTC in Turkish patients.

Age is the strongest predictor of survival in nonmedullary thyroid carcinoma. Some groups report a lower age at diagnosis in familial cases [1,13]. However, we did not report a significant difference in age between familial and sporadic cases, like many others [3,8–12]. This may be related to missed screening following the diagnosis in the other family members. Males seem to be predominant in some series as ours [3], whereas there are others showing similar rates in both genders [1,9,19].

Considering the characteristics of the tumour, the tumour size was generally similar in fPTC cases compared to sPTC cases [4,9,12]. Our findings supported this. However, a study from China reported larger tumour size in familial cases [11]. Multifocality was increased in the familial cases in our study compared to the sporadic ones. There is conflicting data in the literature with regards to multifocality in fPTC patients [3,9,10,20]. 

ETE and LNM are important features of aggressiveness. Our findings revealed increased ETE and LNM in fPTC patients compared to sPTC patients. Many studies reported a significantly higher rate of ETE and LNM in fPTC with regards to sPTC [8-11,13]. In contrast to these findings, some authors have found no association between family history and nodal disease [1,12,20,23]. 

Previous reports on fPTC indicated that recurrence risk was associated with size, number of foci, subtype of the tumour, ETE, vascular invasion and metastasis [8]. There is data showing the diagnosis of PTC in family members to be solely a risk for recurrence compared to sPTC [4,9,11–13,16,17]. This data has not been included in risk stratification yet. Other authors reported no differences in the clinical behaviour of sporadic and familial PTCs [18–21]. In our study, although tendency to multifocality, increased rate of ETE, LNM, and distant metastasis were seen in the fPTC cases. Our comparative analysis between familial and sporadic PTC showed similar recurrence rate. We used American Thyroid Association (ATA) Guideline 2015 to determine the indication for RAI therapy. Having LNM, distant metastasis and ETE were higher in familial group than sporadic group. Thus, the tumours in this group had more risk indicators for RAI therapy. As a result, more patients in the familial group has got RAI and this might have been an explanation why the recurrence and reoperation rates were similar between the familial and sporadic groups. 

The subtype of the tumour is an important factor affecting the risk of recurrence. Our study population consisted mostly of classical subtype of PTC. Considering the nonclassical variants, mostly were nonaggressive types. When the analysis of classical and nonclassical types were made separately, features like distant metastasis were more evident in the familial group regardless of the tumour subtype. 

How the number of affected members is associated with tumour aggressiveness remains unknown. Some studies showed that fPTC with 3 or more affected members have more aggressive features than with 2 affected members [9,11,16,17,27]. However, there is also contradictory data [14,15,23]. In this study, we found that patients in the 3 affected members group were more likely to have distant metastasis and bilateral growth. The recurrence rate of patients with 2 affected family members did not differ from with 3 or more affected family members in our analysis. Our findings were in agreement with several reports [13,16]. However, the numbers of patients with 3 or more affected members were few in our study population to make a definitive conclusion. When the comparison of patients with 3 or more affected family members was made with the sPTC group, ETE was more evident in the sporadic group while distant metastasis was more evident in the fPTC group. Larger patient groups would be needed to elucidate this point.

The optimal treatment for patients with a diagnosis of fPTC is unknown. The majority of authors recommend that patients should undergo total thyroidectomy [23]. In addition, considering that the rate of LNM was higher in familial disease than in sporadic disease, some authors recommend prophylactic central neck dissection [17]. However, not all groups agree [28]. The role of RAI treatment is also currently unclear. In a study from Brazil, with tumours of ≤ 20 mm, with nonaggressive histology, no ETE, no important lymph node involvement, and no evidence of persistent disease after surgery, none of the patients received RAI [28]. No case of recurrence was detected [28]. There are groups that recommend total thyroidectomy followed by postoperative RAI therapy, regardless of tumour size [29]. In our analysis, all patients had total thyroidectomy and the majority of the fPTC cases had received RAI therapy. The indication for RAI treatment were determined by tumour size of >10 mm and the presence of multifocality, ETE, LNM, and distant metastasis. Since all these features were present in the tumours in the fPTC group, RAI therapy had been applied in all of them. 

There are many studies from different countries regarding the character of fPTC. A single centre study in China found that patients with fPTC were more likely to present as large tumours, with multifocality, local invasion, and LNM [11]. In a Japanese study, Ito et al. showed that the occurrence of multifocality, and recurrence rate were significantly higher in their fPTC patients. However, LNM, local invasion, and disease-free survival rate did not significantly differ between familial and sporadic patient groups [23]. A study with 2 different cohorts from Italy and Greece demonstrated that tumours of fPTC patients were more frequently multifocal, tended to have higher recurrence rate, and had worse outcomes when compared with sPTC patients [24]. Another study from Italy also confirmed these findings that fPTC had increased aggressiveness at diagnosis, a higher rate of persistent/recurrent disease, and mortality [10]. However, in Bulgaria, Vidinov et al. showed a statistically significant difference only in tumour size between the familial and sporadic groups [20]. To our knowledge, this is the first study evaluating the clinicopathological and prognostic features of the fPTC patients in Turkish population. The mean postoperative follow-up duration was about 10 years. According to results of our study, it may be that fPTC has more aggressive behaviour with multifocal disease, bilateral disease, extrathyroidal spread, a higher rate of LNM, and distant metastasis. We demonstrated that the recurrence rate of patients with fPTC was the same as patients with sPTC in Turkish population. The unique part of our study is that although the tumours seemed to be more aggressive in the fPTC group compared to the sPTC group, probably the increased application of RAI decreased the recurrences. It may also have regional implications, although at least data from countries close by expressed more aggressive features in the familial forms as in our cases. 

The study included patients who had their surgeries between the dates of 2001 and 2015. Mostly the choices of the operations were usually total thyroidectomy. Recently the recommendations of most of the societies are in favour of less invasive surgeries. The approach might have been different if the surgeries were conducted today, but there is no data in the guidelines regarding the familial cases. By the less invasive surgeries and probably less incline towards RAI therapy, it would probably be easier to observe the recurrence rates.

The study had some limitations. Firstly, the follow-up period had a very great range, from 3 years to 17 years. Given the indolent course of the PTC, this might lead to a heterogeneous follow-up periods of the patients to evaluate the outcomes. The second limitation was retrospective study design. 

In conclusion; our study showed that family history was an important risk factor for more aggressive characteristics with high incidence of multifocality, bilaterality, ETE, LNM, and a higher rate of distant metastasis in Turkish patients with PTC. However, recurrence rates did not differ between the familial and sporadic groups.

## Acknowledgments/Disclaimers

Fundings: Not applicable.

Consent: All of our patients permitted us to use their medical information.
